# PD-L1 is expressed on human platelets and is affected by immune checkpoint therapy

**DOI:** 10.18632/oncotarget.25446

**Published:** 2018-06-08

**Authors:** Verena Rolfes, Christian Idel, Ralph Pries, Kirstin Plötze-Martin, Jens Habermann, Timo Gemoll, Sabine Bohnet, Eicke Latz, Julika Ribbat-Idel, Bernardo S. Franklin, Barbara Wollenberg

**Affiliations:** ^1^ Institute of Innate Immunity, University Hospital, University of Bonn, Bonn, Germany; ^2^ University Hospital Schleswig Holstein, Campus Lübeck, Clinic for Otorhinolaryngology – Head and Neck Surgery, Luebeck, Germany; ^3^ University Hospital Schleswig Holstein, Campus Lübeck, Section for Translational Oncology and Biobanking, Clinic for Surgery, Luebeck, Germany; ^4^ University Hospital Schleswig Holstein, Campus Lübeck, Clinic for Pulmonary Medicine, Luebeck, Germany; ^5^ Department of Infectious Diseases and Immunology, University of Massachusetts Medical School, Worcester, MA, USA; ^6^ German Center for Neurodegenerative Diseases, Bonn, Germany; ^7^ Department of Pathology, University Medical Center Schleswig-Holstein, Luebeck, Germany

**Keywords:** head and neck cancer, biomarkers for PD1-PD-L1 checkpoint therapy, tumor-educated platelets, atezolizumab

## Abstract

Cancer immunotherapy has been revolutionised by drugs that enhance the ability of the immune system to detect and fight tumors. Immune checkpoint therapies that target the programmed death-1 receptor (PD-1), or its ligand (PD-L1) have shown unprecedented rates of durable clinical responses in patients with various cancer types. However, there is still a large fraction of patients that do not respond to checkpoint inhibitors, and the challenge remains to find cellular and molecular cues that could predict which patients would benefit from these therapies. Using a series of qualitative and quantitative methods we show here that PBMCs and platelets from smokers and patients with head and neck squamous cell carcinoma (HNSCC) or lung cancer express and up-regulate PD-L1 independently of tumor stage. Furthermore, treatment with Atezolizumab, a fully humanised monoclonal antibody against PD-L1, in 4 patients with lung cancer caused a decrease in PD-L1 expression in platelets, which was restored over 20 days. Altogether, our findings reveal the expression of the main therapeutic target in current checkpoint therapies in human platelets and highlight their potential as biomarkers to predict successful therapeutic outcomes.

## INTRODUCTION

The tumor microenvironment is remarkably immunosuppressive. Many tumors evade immune cell attack by expressing signalling molecules that trigger fail-safe mechanisms, normally involved in the regulation of uncontrolled inflammation, or autoimmune responses. For example, tumors express programmed death-ligand 1 (PD-L1) which binds to the immune checkpoint protein programmed cell death protein 1 (PD-1) expressed on antigen-specific CD8(+) T cells. Binding of tumor-derived PD-L1 to PD-1 limits the host immune response essentially by switching off T cells that would normally attack cancer cells. Checkpoint inhibition therapies decouple the PD-1/PD-L1 pairing and release the molecular brakes off T cells, to unleash them on tumors [1].

Checkpoint inhibitors have shown unprecedented rates of durable clinical responses in patients with various cancer types, and have now moved to the forefront of cancer research. Since March 2015, several checkpoint inhibitors were granted approval by the US Food and Drug Administration (FDA) to treat a variety of cancers. However, many clinical trials suggest that only a few types of cancer respond to checkpoint inhibition, and only a fraction of patients who are eligible for treatment with checkpoint inhibitors respond to therapy. In most solid cancers only about a third of the patients respond with a prolonged overall survival.

The reasons why some patients respond to PD-L1 blockage, while others do not, are not fully understood. The expression of PD-L1 by cancer cells [2], the quality of T cells circulating in the blood [3], and that infiltrate tumors [4], the mutational rate of cancer cells [5], as well as defects on DNA repair [6], the sensitivity to immune effectors [7], and even the presence of specific microbes in the gut [8–10] have all been associated with improved response to checkpoint inhibitors. With increasing understanding of various cell-to-cell interactions [11] the hunt is on for reliable biological markers (biomarkers) that could effectively flag people who are most likely to benefit from individualized cancer immunotherapy [12].

Hence, identifying tumor or host immune cells that express PD-L1, which set the brakes on T cells, might help to improve the efficacy of immunecheckpoint therapies or indicate novel therapeutic targets. Importantly, although the expression of PD-L1 in tumors may be used as indicators of clinical response, blood-based profiling to understand the mechanisms of PD-1 blockade remains poorly explored. Inspired by this, we performed PD-L1 staining in peripheral blood mononucleated cells (PBMCs) from a cohort of patients with head and neck squamous cell carcinoma (HNSCC). Using flow cytometry, two-photon microscopy and luminex multiplex cytokine arrays, we report here that blood circulating platelets isolated from HNSCC patients up-regulate PD-L1, while PD-L1 is only marginally expressed by platelets from healthy donors. Importantly, cigarette smoking, a key risk factor for HNSCC and lung cancer, was sufficient to up-regulate PD-L1 expression in platelets from cancer-free smokers. Furthermore, we observed that platelet-derived PD-L1 (pPD-L1) is directly affected by Atezolizumab, an anti-PD-L1 based therapy. Treatment of lung cancer patients with the PD-L1 inhibitor atezolizumab caused a decrease of PD-L1 in platelets and PBMCs, without affecting whole platelet counts or white blood cell counts. Our data suggest that rather than being bystander cell fragments, platelets may have active roles in cancer immunotherapy through their expression of PD-L1. The functional significance and predictive value of pPD-L1 for PD-L1 therapies warrants further investigations.

## RESULTS

### Increased PD-L1 expression on PBMCs in peripheral blood of HNSCC patients

In many solid cancers the expression of PD-L1 on tumors has been associated with better response to PD1 and PD-L1 immunotherapy [14]. However, assessment of PD-L1 expression in less invasive blood-based sampling has not been explored widely. Therefore, we assessed PD-L1 fluorescence on PBMCs isolated from healthy donors, or HNSCC patients in different stages of the disease. HNSCC patients were stratified according to their disease status: metastatic (N+), or non-metastatic (N-), based on the occurrence of lymph node metastasis, as well as primary, or recurrent tumors. To evaluate the predictive capacity of PD-L1 on immune cells for the development of HNSCC, we assessed the expression of PD-L1 in PBMCs from patients with smoking habits. Smoking is the number one risk-factor for the development of HNSCC, and has also been associated with increased production of interferon-gamma (IFN-g) [15] a key cytokine that enhances PD-L1 expression in immune cells [16].

**Figure 1:**
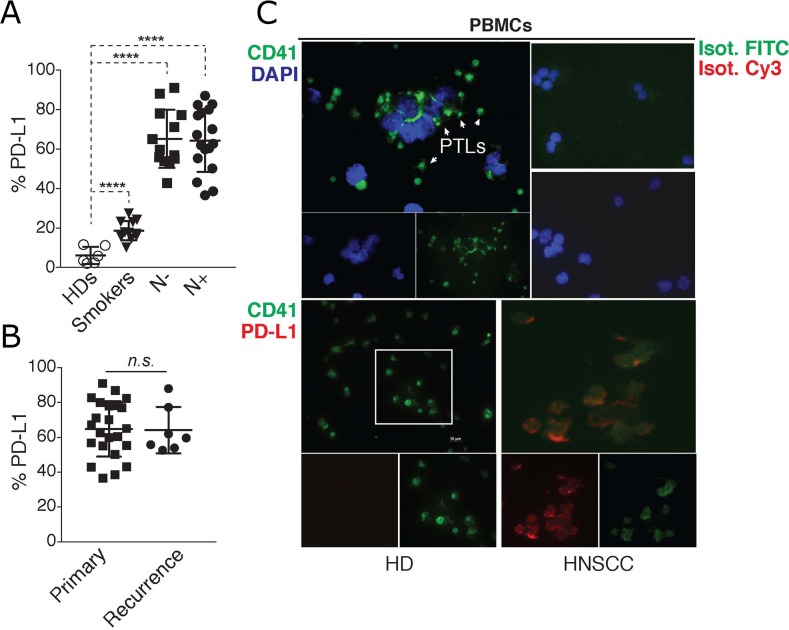
PD-L1 is up-regulated in blood immune cells and platelets of HNSCC patients **(A)** Flow cytometric quantification of % of PD-L1 fluorescence in PBMCs isolated from healthy donors (HDs, n = 6), smokers (n = 12), and HNSCC patients with (N+, n = 16), or without (N−, n = 7) lymph node metastasis. Statistical analysis was performed by Ordinary one-way ANOVA, Tukey’s multiple comparisons test. ^****^ *p < 0.0001*. **(B)** N+ and N− HNSCC patients in (A) were further stratified accordingly to the tumor incidence as primary, or recurrent cancer. Graphs show the % of PD-L1 fluorescence in PBMCs in relation to tumor incidence. Unpaired *t* test was used to calculate the differences. n.s. *p = 0.9230*. **(C)** **Top:** Two-photon microscopy (TPEF) imaging of cytospin slides containing PBMCs from a HNSCC patient stained with the platelet marker CD41. Nuclei were stained with (DAPI). **Bottom:** TPEF imaging of PD-L1 and CD41 staining on cytospin slides containing PBMCs from a healthy donor, or a HNSCC patient, showing PD-L1 expression on platelets (CD41+ cells). Data is representative of 10 different experiments.

In agreement with previous observations [17], PD-L1 expression was increased in circulating immune cells of HNSCC patients, irrespectively of their metastatic status (Figure 1A). HNSCC patients (N+ and N-) were further stratified according to the incidence of tumors, as primary and recurrent cancers (Figure 1B). PD-L1 was similarly expressed on PBMCs from these patients irrespectively of their tumor incidence. Those data suggest that the presence of locoregional or distant metastases did not influence the quantity of PD-L1 expression on immune cells.

The PBMC fraction is comprised of different immune cells which express PD-L1 [18]. To locally associate PD-L1 expression in the PBMC population, we performed two-photon fluorescence microscopy (TPEF) in cytopsin slides containing fixed PBMCs from healthy donors or HNSCC patients. TPEF imaging of PBMCs from these patients revealed the presence of a large quantity of platelets, which surprisingly, were also found to express PD-L1 (Figure 1C). Importantly, platelets present in PBMC preparations from healthy donors, were mostly negative for PD-L1 (Figure 1C). Although PD-L1 has been reported on immune cells [16], these findings are unprecedented as there are no reports of its expression on human platelets. Thus, we investigated more closely the expression of platelet PD-L1 (pPD-L1) in HNSCC cancer.

In the same HNSCC cohort as above we examined the expression of PD-L1 in cells from the tumors, or in the immune cells recovered from solid tumors (tumor infiltrating cells). Most samples expressed PD-L1 on very low levels within both cell populations (around 1%). No correlations were found between PD-L1 expression in the tumor, or on tumor infiltrating cells and PD-L1 in peripheral blood cells (data not shown).

### Increased PD-L1 expression on purified platelets from HNSCC patients

**Figure 2:**
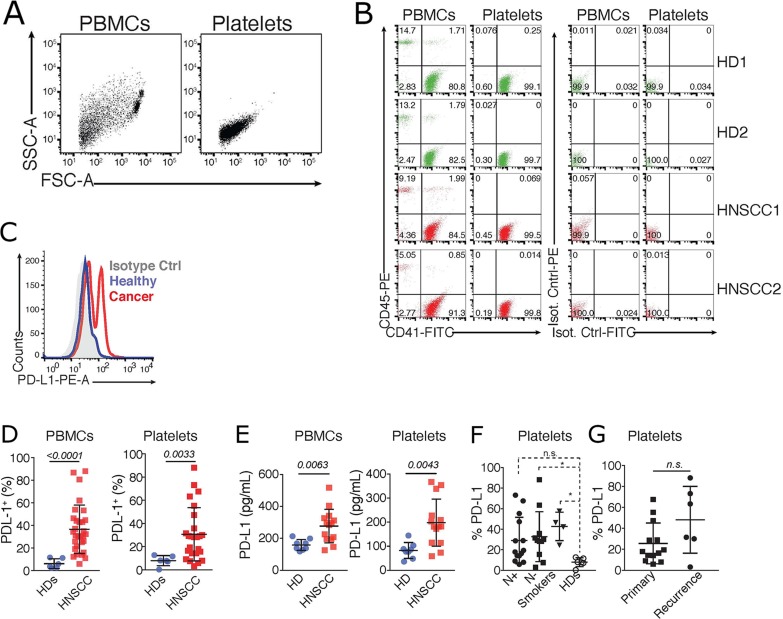
PD-L1 is upregulated in blood platelets from HNSCC patients **(A)** Flow cytometric analysis showing the scatter characteristics and the gating strategy to assess the purity of platelets. Data is representative of >30 independent experiments with different donors. **(B)** Cells were further gated by their expression of the leukocyte (CD45) and platelet (CD41) markers, compared to staining with IgG matched isotype controls. Data shows two representative healthy donors (HD) and HNSCC patients. **(C)** Representative histogram of the fluorescence of PD-L1, or an IgG matched isotype control, on PBMCs and platelets isolated from a HD, and a HNSCC patient. **(D)** Cummulative percentages of PD-L1 on PBMCs, and platelets (PD-L1^+^CD41^+^) isolated from HDs (n = 6, same as in Figure 1A), and HNSCC patients (n = 37). **(E)** Luminex Cytokine Array comparing PD-L1 levels in PBMCs and platelets isolated from HDs (n = 8) and HNSCC patients (n = 14). Statistical differences were calculated using Unpaired *T* test. P values are indicated. **(F)** Flow cytometric quantification of % of PD-L1 fluorescence in purified platelets from healthy donors (HDs, n = 6), smokers (n = 12), and HNSCC patients with (N+, n = 16), or without (N−, n = 7) lymph node metastasis. Statistical analysis was performed by Kruskal-Wallis, Dunn’s multiple comparisons test. ^*^ *p < 0.05*. **(G)** N+ and N− HNSCC patients in **F** were further stratified accordingly to the tumor incidence as primary or recurrent cancer. Graphs show the % of PD-L1 fluorescence in purified platelets in relation to tumor incidence. ManN−Whitney test was used to calculate the differences. n.s. P values are indicated.

Next, we employed different methods to assess the dynamics of PD-L1 expression in highly purified platelets from healthy donors and HNSCC patients. For this, platelets were purified from peripheral blood (see methods) and their purity was assessed using leukocyte (CD45) and platelet markers (CD41), as well as matching isotype controls. Flow cytometric assessment revealed that platelet preparations were mostly free of contaminating leukocytes (Figure 2A and 2B). Then, we assessed the protein expression of PD-L1 on the surface of platelets from healthy donors (HDs), or HNSCC patients by flow cytometry. PBMCs from the same donors were used as comparison. We found a significant increase in PD-L1 on platelets and PBMCs of HNSCC patients compared to healthy donors (Figure 2C-2D). These fluorescence measurements were further confirmed by a luminex cytokine plex performed on whole cell lysates of PBMCs and purified platelets from HNSCC patients (Figure 2E). In line with what we observed with PBMCs (Figure 1), PD-L1 was similarly expressed on platelets isolated from HNSCC patients that were stratified based on the incidence of their tumors, or their metastatic stage (Figure 2F).

### pPD-L1 is affected by treatment with the immune check-point inhibitor atezolizumab

**Figure 3:**
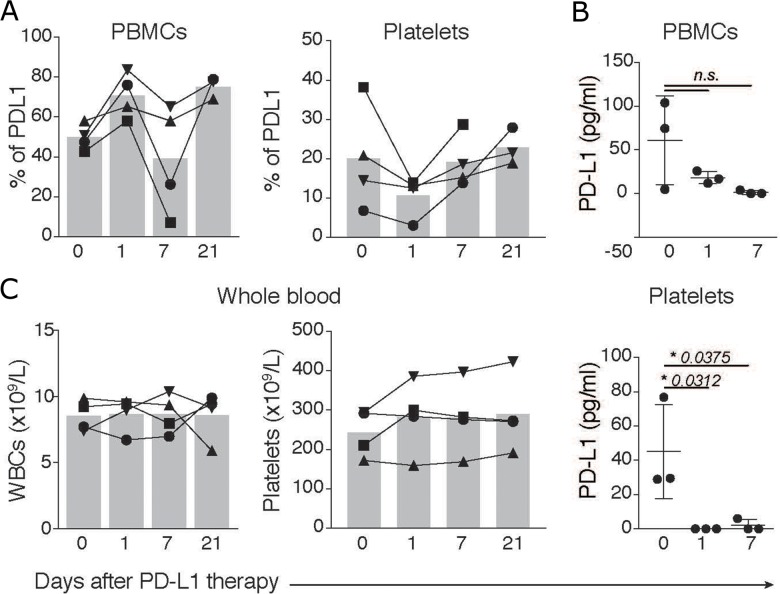
PD-L1 in platelets and PBMCs is affected by atezolizumab **(A)** Flow cytometric assessment of the percentages of PD-L1 fluorescence in PBMCs, or platelets (gated on CD41^+^PD-L1^+^ events) of lung cancer patients (n = 4) before (day 0) or at 1, 7 and 21 days after therapy with 1,200 mg of atezolizumab. **(B)** Luminex Cytokine Array comparing PD-L1 levels in PBMCs and purified platelets from lung cancer patients before and after several days post therapy as in **A**. **(C)** Total leukocyte and platelet counts in peripheral blood of lung cancer patients before (day 0) or at 1, 7 and 21 days after therapy. Differences were calculated using Ordinary one-way ANOVA, Tukey’s multiple comparisons test.

Next, we speculated that expression of PD-L1 on platelets might render them susceptible targets of antibody-based anti PD-L1 therapies. Indeed, thrombocytopenia is a fairly common side-effect of immune checkpoint inhibitors [19]. And, recently, two cancer drug trials were paused after evidences of bleeding disorders in patients receiving anti-PDL-1 therapy (Kestrell Study -https://uk.reuters.com/article/us-astrazeneca-cancer/astrazeneca-pauses-two-cancer-drug-trials-enrolment-due-to-bleeding-idUKKCN12R2D4). To investigate this hypothesis, we assessed total platelet counts and PD-L1 expression in platelets purified from lung cancer patients (n = 4) before and several days after treatment with the PD-L1 targeting antibody Atezolizumab in a routine clinical application. Lung cancer patients received a flat dose of 1200 mg atezolizumab in a 3-week regimen. The therapy was well tolerated. Besides routine control of white blood cell counts (WBCs) the values of PD-L1 expressing cells were monitored throughout therapy. Assessments of PD-L1 expression on PBMCs and blood purified platelets revealed that while atezolizumab did not significantly affect the PD-L1 expression on PBMCs (Figure 3A and 3B), the amount of PD-L1 expressing platelets diminished in the blood of treated patients in the first 7 days of therapy (Figure 3B). Importantly, atezolizumab did not significantly affect the clinical total platelet or white blood cell count (Figure 3C), suggesting that PD-L1 inhibition did not affect free platelets, but might have targeted platelets complexed to immune cells. PD-L1 expression on PBMCs and platelets were reconstituted after 21 days of therapy, consistent with the beginning of the next atezolizumab cycle. Altogether, our data indicate that blood platelets are a dynamic source of PD-L1, which may have potential effects on cancer immunotherapy.

After 3 cycles of therapy, a follow up examination by CT scan revealed no increase in tumor burden, but rather a slight regression. Although atezulizumab therapy was halted in one patient due to occurence of severe allergic reactions, this patient showed a stable disease after 3 cycles of therapy.

Our findings indicate that anti-PD-L1 therapy targets PD-L1 expressed on platelets. This observation could potentially arise from competition for epitope binging between Atezolizumab and our PE-labeled anti-PD-L1 antibody. To exclude this possibility we purified platelets from a HNSCC patient and incubated them ex-vivo with clinical concentrations of Atezolizumab (as well as 10 fold higher dosages). Atezolizumab treated platelets were then co-stained with the anti-PD-L1 used in our study. Importantly, pre-incubation of freshly isolated HNSCC platelets with Atezolizumab did not influence the staining of PD-L1 *in vitro* (Data not shown). Furthermore, PD-L1 expression was not affected on PBMCs before vs after therapy. Altogether, these findings indicate that epitope binding competivity does not account for the observed effects of Atezolizumab on PD-L1 expression in treated patients.

## DISCUSSION

A long-standing question in cancer immunotherapy is how to identify the most suitable patients that would benefit from immune checkpoint inhibitors. Despite the successes of checkpoint inhibitors in several cancers, including HNSCC [20, 21], there are still a substantial proportion of patients that do not respond to therapy. Hence, considerable research efforts are now directed to identify routinely available blood and clinical markers that may predict response to therapy. Our study provides the first report of PD-L1 expression in circulating blood platelets and introduces them as potentially novel and noN−invasive biomarkers in head and neck squamous cell carcinoma (HNSCC), with yet unexplored implications for checkpoint inhibition therapies. These observations were also extended to other cancer types, as we also found increased PD-L1 expression on platelets from patients with lung cancer. In our patient cohort, PD-L1 expression was not correlated with disease stage, the occurence of lymph node metastasis, or with the incidence of the tumor (primary vs recurrent). Indeed, platelet-PD-L1 (pPD-L1) levels were comparably high in all cancer cases compared to healthy donors. Interestingly, we found that cancer-free individuals with smoking habits displayed an intermediate phenotype, with significantly higher PD-L1 expression in both PBMCs and purified platelets, compared to healthy donors.

The presence of infiltrating immune cells, mainly CD8+ T cells, both at the invasive tumor margin and inside tumors is an essential element to predict better responses to immune checkpoint therapies [4]. The core of solid tumors is not easily assessed by immune cells, and the few infiltrating immune cells that gain access, must battle this immunosuppressive environment to kill the target cancer cells. PD-L1 expression on immune cells infiltrating the HNSCC are thought to be favorable prognostic factors for resected HNSCC, highlighting the importance to distinguish between PD-L1 expression on tumor cells (TC) and tumor-infiltrating immune cells (IC) [17]. We have found that the elevated PD-L1 expression in the peripheral blood did not correlate with the PD-L1 expression within the tumor environment, or in the immune cells recovered on from the tumor. This might explain previous reports of better response to check point inhibitors in patients with low or negative PD-L1 expression in tumors. PD-L1 expression in the peripheral blood might be a determining factor to predict the outcome of check-point immunotherapy. In this context, platelets arise as potential immune players in cancer therapy. There are approximately one trillion platelets circulating in the blood of a healthy adult (150 - 400 × 109/L). These cells permeate through the small capillary vessels, and can reach deep areas in the tissues, including the core of a tumor, which is usually refractory to most immune cells. Tumor infiltrating platelets can incorporate tumor-associated molecules, a phenomenon known as “education”, and convey accurate information about cancer signatures [22, 23]. Indeed, this and other features of platelets have been successfully employed as biomarkers for early detection of tumors [24, 25]. The close interactions of platelets and cancer cells is rapidly becoming a fruitful area of scientific investigation and is currently being explored therapeutically. A recent study conjugated anti-PD-1 engineered monoclonal antibodies to the surface of platelets, which were used to deliver anti-PD-1 therapies directly into tumors. In a pre-clinical model of melanoma, platelet activation caused effective release of anti-PDL1 by platelet-derived microparticles and prolonged survival after surgery by reducing the risk of cancer regrowth and metastatic spread [24, 25]. On the other hand, platelets have also been involved in tumor metastasis by different mechanisms [26]. Platelet-derived TGFβ and direct platelet-tumor cell contacts mediate the transition of cancer cells into an invasive mesenchymal-like phenotype and enhanced metastasis *in vivo* [27]. Platelets were also reported to shield circulating tumor cells from recognition through the immune system [28].

Importantly, we found that PD-L1 expression is up-regulated in platelets and PBMCs independently of tumor stage (N−, stage I and II; N+ stage III and IV), and is already enhanced in platelets and PBMCs from smokers, which are under increased risk to develop HNSCC or lung cancer. These findings imply that pPD-L1 may have powerful predictive power and, as previously reported [22], could be an useful biomarker for early stage cancers. The expression of PD-L1 on platelets from cancer patients also raises the possibility that pPD-L1 could interfere with immune checkpoint therapies, likely by competing with therapeutic antibodies directed against PD-L1 on cancer cells. This hypothesis, though not tested experimentally, is supported by observations that high platelet counts are associated with poor prognosis in several types of cancers [29], and were reported to impair the response to primary tyrosine kinase inhibitor therapy for metastatic renal cell carcinoma [30].

The use of liquid biopsies for the monitoring of checkpoint relevant drugs is completely new and unexplored. Our findings show that whole blood counts (WBCs, Figure 3C) are not influenced by atezolizumab therapy and therefore are not reliable to assess clinical response to immune checkpoint therapy. With the search for blood-based biomarkers on the rise, our study highlights the importance of considering platelets for the assessment of PD-L1 expression, which would also help to select patients suitable for PD-1 therapies. The FDA has set high levels of PD-L1 expression in the primary cancer tissue as a criterion for treatment with Pembrolizumab, a PD-1 blocking antibody, for certain cancers, e.g. lung cancer. Furthermore, insurance companies rely on the percentage of PD-L1 expression in the primary cancer to include patients and cover expenses of clinical treatment with PD1 or PDL1 inhibitors. In HNSCC PD-L1 expression in cancer tissue shows only modest utility in predicting such benefit. Many HNSCC patients that experienced a partial or complete remission displayed a mere 10% positivity of PD-L1, some of them were even PD-L1 negative before therapy [21]. We hope that new studies with larger cohorts will validate and correlate the pPD-L1 with clinical response rates. If pPD-L1 has biomarker potential, it could also help to make personalised therapy easier, and change the current flat dose regimen (where equal doses are administered to all patient) to a more individualised concept based on, platelet counts, and pPD-L1 levels in the blood.

### Study limitations

Although we could sucessfully purify blood platelets from most contaminating leukocytes, the PBMC fraction was largely contaminated with platelets. Thus, it is not yet possible to account for how much of the PD-L1 expression detected on PBMCs is derived from complexed platelets.

Furthermore, although we observed decreased PD-L1 expression on platelets from patients treated with atezolizumab, the therapy did not affect whole platelet blood counts. Two possible reasons for these findings are: i) Atezolizumab targets PD-L1 on platelets complexed to immune cells, which are not included on total platelets counts assessed with automatic whole blood counters. ii) The low sample size (n = 4) of the cohort for before vs after atezolizumab.

Our study also raises new questions: what are the mechanisms that drive the up-regulation of PD-L1 in circulating blood platelets? Are platelets educated at the tumor site to up-regulate pPD-L1, or do tumors secrete factors that induce megakaryocytes to release platelets containing pPD-L1? These hypotheses need further experimental validation. For example, immunoprotein profiling of megakaryocytes from HNSCC patients, or incubation of megakaryocytic cell lines with tumor conditioned medium will aid in the investigations.

Furthermore, what are the functions of PD-L1 expressing platelets in tumor development, or in immunotherapy? Finally, the suitability of pPD-L1 as a general tumor biomarker still warrants investigations in larger cohorts and in different cancer types.

## MATERIALS AND METHODS

### Patients characteristics

This study was approved by the ethics committee of the University of Lübeck (Az16-278/2017). All patients enrolled have signed an informed written consent, and were educated about the aims of the study and the use of their samples. Blood samples were collected from healthy donors (n=8), healthy voluntaring smokers (n=12), head and neck squamous cell carcinoma (HNSCC) patients from all anatomical regions (HNSCC stage I:4; stage II: 2; stage III 7, stage IV:17) and lung cancer patients (stage IV:4). All patients were selected randomly from July to December 2017 and received a routine clinical workup for diagnosis and guideline adapted treatment. Blood samples were drawn prior to therapy. Tumor stage was evaluated for every patient by CT scans and clinical examination.

### Atezolizumab treatment regime

According to new treatment standards, the four patients with lung cancer were treated with Atezolizumab (trade name Tecentriq) a fully humanized, engineered monoclonal antibody of IgG1 isotype against the protein programmed cell death-ligand 1 (PD-L1). All four patients received 1200 mg i.v. flat dose in a three weekly regimen. Clinically therapy was tolerated very well by all four patients without relevant side effects.

### Platelet and PBMC isolation from human blood

Human platelets were isolated essentially as previously described [13], with slight modifications. In brief, venous blood was drawn into S-Monovette^®^ 9NC collection tubes. The blood was centrifuged for 5 minutes at 330g without brake to obtain platelet-rich plasma (PRP). All following centrifugation steps were performed without brake and in the presence of 200nM PGE1 to inhibit platelet activation. PRP was transferred to a new tube and diluted 1:1 with phosphate-buffered saline (PBS) to reduce leukocyte contamination and centrifuged for 10 minutes at 240 x g. Platelets were pelleted by centrifugation at 430 x g for 15 minutes and washed once with PBS, before they were lysed in RIPA buffer (1% Igepal CA-630, 0.5% Natrium Deoxycholat, 0.1% SDS in 1x PBS). PBMCs were isolated from the remaining blood fraction after taking off the PRP by density gradient centrifugation in Ficoll-Paque PLUS. After washing the PBMCs twice with PBS, cells were also lysed in RIPA buffer containing protease and phosphatase inhibitors. The purity of the isolated platelets and PBMCs was assessed by flow cytometry using CD45 (leukocyte) and CD41a (platelet) markers.

### Purity assessment of the isolated cells by flow cytometry

Samples of isolated platelets and PBMCs were analysed for purity after each experiment. Cells were blocked with 1:10 human Fc blocking reagent for 10 minutes at room temperature (RT) before samples were stained with fluorochrome-conjugated monoclonal anti-human Ig antibodies against CD41a and CD45 for 30 minutes in the dark. Cells were washed and resuspended in flow cytometry buffer (1% FCS in PBS) for analysis. Compensation beads (OneComp eBeads) and isotype controls were prepared in the same way. Cell viability was confirmed using by Annexin V and propidium iodide staining (BD Biosciences, Heidelberg, Germany). Flow cytometry was performed with a FACS Canto A flow cytometer and data were analysed using FACS Diva software 6.0 (BD Biosciences, Heidelberg, Germany) and the FlowJo software (Tree Star). The applied gating strategy was based on doublet discrimination and isotype-matched control antibodies.

**Table:** List of reagents

REAGENT or RESOURCE	SOURCE	IDENTIFIER
**Antibodies**		
Anti-human CD41a FITC (HIP8)	eBioscience	Cat#11-0419-42
Anti-human CD45 PE (2D1)	eBioscience	Cat#12-9459-42
Anti-human PD-L1 PE	Biolegend	Cat#329706
Anti-human CD41	Biolegend	Cat#303704
Anti-human PD-L1	Abcam	Cat#ab58810
donkey anti rabbit Cy3 secondary antibody	Invitrogen	Cat#A21206
goat anti rabbit FITC secondary antibody	Jackson ImmunoRes	Cat#111-165-003
FcR Blocking Reagent, human	Miltenyi Biotech	Cat#130-059-901
OneComp eBeads	eBioscience	Cat#01-1111-42
**Chemicals, Peptides and Recombinant Proteins**		
Ficoll-Paque PLUS	GE Healthcare	Cat#17-1440-03
GibcoTM RPMI 1640 Medium	Thermo Fisher	Cat#21875091
GibcoTM Dulbecco's Phosphate Buffered Saline (PBS)	Thermo Fisher	Cat#14190144
Fetal Calf Serum (FCS)	Invitrogen	
Prostaglandin E1 (PGE-1)	Sigma-Aldrich	Cat#P5515-1MG
**Critical Commercial Assays**		
S-Monovette 9NC tubes	Sarstedt	Cat#01.1606.001
Immuno-Oncology Checkpoint 14-Plex Human ProcartaPlex™ Panel 1	ThermoFisher Scientific	EPX14A-15803-901
**Software and Algorithms**		
GraphPad Prism 7	GraphPad	N/A
FlowJo 10.4	FlowJo	N/A

### Cytospins of PBMC/Immunohistochemistry

Peripheral Blood (7.5 mL) was mixed with equal volume of ice-cold PBS and carefully transferred onto a 15 ml layer of cold Ficoll-solution. and centrifuged at 15 °C for 20 min at 800 × g. The upper plasma layer was carefully removed and discarded. The interphase (containing PBMCs) was transferred into a fresh 50-ml tube. Cold PBS was added to a total volume of 25 mL and samples were centrifuged at 4 °C for 5 min at 300 × g. The supernatant was discarded and cells resuspended in PBS. PBMC cytospins were prepared using 100 μL of cell suspension, which was centrifuged in a cytofuge for 4 min at 800 rpm. Cytospins were air-dried in the dark overnight. Slides were covered with 20% acetone for 10 minutes for fixation and permeabilization, followed by incubation with the specific primary antibodies anti-CD41 and anti-PD-L1, or IgG matched isotope controls over night at 4°C. After washing with PBS the secondary antibodies goat anti-rabbit Cy3 and goat anti-rabbit FITC were incubated for 45 min, respectively. The slides were rinsed three times in PBS. Nuclei were stained with DAPI (1 μg/mL, Roche Diagnostics, Mannheim, Germany). The samples were rinsed three times for 5 minutes in PBS and embedded in Fluoromount G (Southern Biotechnologies Associates, Birmingham, Alabama, USA). Samples were imaged using Two-photon microscopy (TPEF).

### Luminex multiplex cytokine measurements

Multi-cytokines in platelet and PBMC lysates were measured with an Immuno-Oncology Checkpoint 14-plex ProcartaPlex^TM^ bead assay from ThermoFisher and measured on a MAGPIX instrument using Luminex xMAP Technology. The analysis was performed according to the manufacturer's instructions with slight modifications. The standard stock vial was diluted in 900μl in total and a 1:2 dilution series was used in the assay. Similarly, 200 μL of samples and standard were loaded onto the 96-well plate before overnight incubation at 4°C. Results were analyzed using GraphPad Prism Version 7.0f.

### IHC staining for PD-L1 in HNSCC tumors

The tissue samples were formaliN−fixed and paraffin embedded (FFPE) (4% buffered formalin, BÜFA, Hude, Germany; Paraffin, Leica Microsystems, Wetzlar, Germany). 4 μm thick slices were cut off from the paraffin blocks by microtome and placed on Super Frost glass slides (Menzel, Braunschweig, Germany). To verify staining success a positive control tissue (placenta) was placed on each slide.

Immunohistochemistry (IHC) was performed using an automated staining platform (Ventana, Roche, Tuscon AZ, USA). We used the E1L3N clone of PD-L1 manufactured by Cell Signalling (Danvers MA, USA,). It was diluted 1:50 and pretreated by Tris-EDTA (pH 8,4, Ventana). An OptiView HRP Detection Kit (Ventana) were used according to the manifacturer's instructions for the visualization of the primary anti PD-L1 antibody.

The CD61 stained tissue slides were examined by a board certified histopathologist (JRI) using Olympus BX50 microscope (Olympus, Hamburg, Germany). The percentage of positive stained tumor cells and the area of positive tumor immune cells were evaluated for each slide.

### Statistical analysis

Statistical analyses were performed with GraphPad Prism Version 7.0f. Data are presented as symbols, each symbol representing individual donors. The mean and standard deviation (SD) is shown when three or less, or mean and standard error (SEM) when four or more biological replicates are represented. The differences between groups were determined after testing for Gaussian distribution (normality tests), and applying parametric (*T* test), or noN−parametric 1-way Anova, Tukey's multiple comparisons test: p < 0.05 (^*^), p < 0.01 (^**^), and p < 0.001 (^***^). Additional statistical details are given in the respective figure legends, when appropriate.
